# Heterologous Expression of *AtWRKY57* Confers Drought Tolerance in *Oryza sativa*

**DOI:** 10.3389/fpls.2016.00145

**Published:** 2016-02-11

**Authors:** Yanjuan Jiang, Yuping Qiu, Yanru Hu, Diqiu Yu

**Affiliations:** ^1^Key Laboratory of Tropical Plant Resources and Sustainable Use, Xishuangbanna Tropical Botanical Garden, Chinese Academy of SciencesKunming, China; ^2^National Plateau Wetlands Research Center, Southwest Forestry UniversityKunming, China

**Keywords:** *At*WRKY57, drought tolerance, *Oryza sativa*, Stress, ROS

## Abstract

Drought stress is a severe environmental factor that greatly restricts plant distribution and crop production. Recently, we have found that overexpressing *AtWRKY57* enhanced drought tolerance in *Arabidopsis thaliana*. In this study, we further reported that the *Arabidopsis* WRKY57 transcription factor was able to confer drought tolerance to transgenic rice (*Oryza sativa*) plants. The enhanced drought tolerance of transgenic rice was resulted from the lower water loss rates, cell death, malondialdehyde contents and relative electrolyte leakage while a higher proline content and reactive oxygen species-scavenging enzyme activities was observed during stress conditions. Moreover, further investigation revealed that the expression levels of several stress-responsive genes were up-regulated in drought-tolerant transgenic rice plants, compared with those in wild-type plants. In addition to the drought tolerance, the *AtWRKY57* over-expressing plants also had enhanced salt and PEG stress tolerances. Taken together, our study indicates that over-expressing *AtWRKY57* in rice improved not only drought tolerance but also salt and PEG tolerance, demonstrating its potential role in crop improvement.

## Introduction

Drought is a critical abiotic stress that severely restricts crop production (Zhu, 2002). With the process of evolution, plants have gained a variety of strategies with the purpose of avoiding drought stress by reducing water loss or increasing water uptake. Nevertheless, other strategies need to protect plant cells from damage when water is exhausted and tissue dehydration unavoidable (Verslues et al., 2006). Additionally, the molecular, cellular, and whole-plant levels strategies should be coordinated to adapt to drought stress (Yu et al., 2008).

Under drought- or salt-stress conditions, plants accumulate reactive oxygen species (ROS) (Verslues et al., 2006). In living cells, ROS such as superoxide, hydrogen peroxide (H_2_O_2_), and hydroxyl radicals are generated as harmful substances via aerobic metabolism. Through partially reduced or activated derivatives of oxygen, ROS can destroy DNA, proteins and carbohydrates, resulting in cell death (Mittler et al., 2004). A master level of ROS gives rise to the oxidation of biomolecules, such as lipids, nucleic acids and proteins, which caused cellular damage. When CO_2_ fixation is restricted under environmental stress conditions, the photosynthetic electron transport system generates ROS (Asada, 1999). To defend oxidative stress, organisms have evolved an effective system to protect themselves. For example, numerous stress-related genes were induced by ROS in response to oxidative stress in these defensive systems (Demple and Amabile-Cuevas, 1991; Gasch et al., 2000; Desikan et al., 2002). As higher plants have the ability to coordinately regulate multiple antioxidant genes, they are much tolerant to oxidative stresses.

Normally, the maintenance of routine homeostasis is achieved through the ROS-scavenging system in plant cells, which is mainly mediated by enzymatic defenses, including superoxide dismutase (SOD), catalase (CAT), and peroxidases (POX) (Mittler et al., 2004). Generally, SODs, which catalyze the dismutation of superoxide into oxygen and H_2_O_2_, provide the first line of defense against ROS in various subcellular compartments, such as chloroplast, mitochondria and cytosol (Raychaudhuri and Deng, 2000). The physiological role of CAT is to break down H_2_O_2_ in the cell (Scandalios, 2002). Therefore, increased CAT activity would result in H_2_O_2_ degradation. PODs are a group of enzymes that catalyze the oxidation of many substrates (e.g., phenolic compounds) at the expense of H_2_O_2_ (Asada, 1987). The increased activity of these enzymes would decrease ROS levels. Recent reports have demonstrated that transgenic rice plants with enhanced ROS-scavenging abilities had improved drought tolerance (Ouyang et al., 2010; Zhang et al., 2011). For example, *OsSIK1* functions in stress signaling through scavenging and detoxification of ROS. In *OsSIK1*-overexpressing plants, the high levels of POD and CAT enzymes resulted to low levels of H_2_O_2_ (Ouyang et al., 2010). Similarly, the improved drought tolerance of *HRF1*-overexpressing transgenic rice plants was partially resulted from the increased ROS-scavenging activities (Zhang et al., 2011).

Under environment stress conditions, the stress-related proteins not only function in protecting cells from damage but also regulate the expression of downstream genes for signal sensing, perception and transduction (Kreps et al., 2002; Seki et al., 2002). These proteins can be classified into two groups. The first group protein plays a crucial role to avoid cellular injury, such as detoxification enzymes, Late Embryogenesis Abundant (LEA) proteins, and the key enzymes for osmolyte biosynthesis (Kreps et al., 2002; Seki et al., 2002). The second group includes numerous transcription factors involved in further regulation of transcriptional control and signal transduction. The CBF/DREB factor, Basic Leucine Zipper families, CUC transcription factor, NAM, plant nuclear factor Y (NF-Y) B subunits, zinc finger and ATAF, belong to this group (Umezawa et al., 2006; Nelson et al., 2007; Takasaki et al., 2010). Studies on these transcription factors will contribute to uncover the respect for commercially improving drought tolerance in crops through genetic engineering.

The WRKY family consists of 74 and 102 members in *Arabidopsis thaliana* and *Oryza sativa*, respectively (Eulgem et al., 2000; Wu et al., 2005); and majority of them play critical roles in biotic and abiotic stress responses (Eulgem and Somssich, 2007; Miller et al., 2008). Recently, increasing evidences confirmed that numerous of *WRKY* genes are involved in drought stress. For example, ABO3/WRKY63 plays a key role in plant responses to ABA and drought stress (Ren et al., 2010). Overexpression of a stress-induced *OsWRKY45* significantly confer drought tolerance in *Arabidopsis* and rice (Qiu and Yu, 2008; Tao et al., 2011). Especially, our previous study demonstrated that overexpression of *AtWRKY57* improved drought tolerance by directly targeting the promoter sequences of *NCED3* to increase the content of ABA in *Arabidopsis* (Jiang et al., 2012). These evidences give us a hypothesis that the improvement of plant drought tolerance might be realized through gene manipulation approaches. To test this hypothesis, we further over-expressed *AtWRKY57* in rice and demonstrated that the stress tolerance of the transgenic rice under drought conditions was significantly improved. 3,3′-Diaminobenzidine (DAB) and nitro blue tetrazolium (NBT) staining analyses showed that the ROS levels in transgenic lines were lower than in control plants after drought-stress treatment. Consistent with the low ROS levels, the antioxidative enzyme activities were also enhanced in the transgenic lines. Moreover, high expression levels of stress-responsive genes also supported the drought tolerance in transgenic lines. Overall, our results indicated that the over-expression of *AtWRKY57* in rice conferred the adaptation of rice to drought tolerance by reducing ROS damage and up-regulating the expression of stress-responsive genes.

## Materials and Methods

### Construction and Transformation of *AtWRKY57* in Rice

The full-length cDNA sequence of *AtWRKY57* was obtained from *Arabidopsis* using the same method as described in our previous study (Jiang et al., 2012). The full coding sequence of *AtWRKY57* was cloned into pUN1301 in the sense orientation behind the Ubiquitin promoter. Then the T-DNA was transformed into ZH11 (*Oryza sativa* L. ssp. *japonica* cv. Zhonghua11) via the *Agrobacterium tumefaciens*-mediated method (Hiei et al., 1994). After transformation, the calli were selected from half-strength (MS) medium containing 100 μg/ml hygromycin. Seedlings with hygromycin-resistant were transplanted to soil in a growth chamber.

### Plant Growth Conditions

The sterilized *Oryza* seeds sowed on medium and kept in a growth chamber at 22°C under long-day conditions [16 h light/8 h dark cycles]. One week generation, seedlings were then transplanted in soil and half-strength MS medium supplemented with 1.5% (W/V) sucrose for drought stress, NaCl and PEG treatments. The soils are commonly used loam, mixed 50% humus soil, 30% coconut tree branny, 20% red clay.

### Drought-Tolerance Assays

Drought-tolerance assays were performed using 4-week-old plants. The transgenic rice and control seedlings were transplanted in the same pot and treated with drought stress by withholding water for 20 days. Three independent pots repeated at the same time and a representative result displayed. Three independent experimental replications were conducted.

To evaluate the water loss rates, flag leaves were detached from the plants and weighed at designated time intervals at room temperature. The proportion of fresh weight lost was calculated based on the initial plant weight. At least three biological replicates for each sample were used for the calculation.

### Trypan Blue, DAB and NBT Staining

For DAB staining, leaf sections of approximately 5 cm in length were cut and soaked in a 1% solution of DAB in 50 mM Tris-HCl buffer (pH 6.5). After 30 min vacuum infiltrating, the immersed leaves were incubated in the dark for 20 h at room temperature. And then the leaves were bleached by bath in boiling ethanol until the brown spots appeared clearly. The area of brown spots are represented the DAB reaction degree to H_2_O_2_.

Leaf sections of approximately 5 cm in length were excised to detect superoxide accumulation by a 0.1% solution of NBT in 10 mM potassium phosphate buffer (pH 7.8) as described previously (Fitzgerald et al., 2004). After 15 min vacuum infiltrating, the immersed leaves were incubated overnight at room temperature. After incubation, the leaves were fixed and cleared in alcoholic lacto-phenol (2:1:1, 95% ethanol:lactic acid:phenol) at 65°C for 30 min, rinsed with 50% ethanol, and then rinsed with water. When NBT interacts with superoxide, a blue precipitate forms is visible in leaves.

### Proline (Pro) Content, Malondialdehyde (MDA) Content, and Electrolyte Leakage Measurements

The proline concentration was determined as described (Bates, 1973). Approximately 0.5 g of transgenic and control leaf segments were homogenized in 10 ml 3% aqueous sulfosalicylic acid and centrifuged at 3,000 × *g* for 20 min. 2 ml of supernatant was reacted with 2 ml acid ninhydrin and 2 ml glacial acetic acid in a test tube at 100°C for 1 h, cooled on ice, and the absorbance at 520 was measured. L-Pro was used as a standard to calculate the proline concentration.

The MDA content was determined as described (Heath and Packer, 1968) with slight modifications. Approximately 1 g of transgenic and control leaf segments were homogenized in 10 ml of 10% trichloroacetic (v/v) and centrifuged at 5,000 × *g* for 10 min. 2 ml of supernatant was reacted with 2 ml thiobarbituric acid in a test tube at 100°C for 15 min, quickly cooled on ice, and the absorbance at 532 was measured. The MDA content was confirmed using the extinction coefficient of 155 nM^-1^ cm^-1^, and expressed as nmol g^-1^ FW.

The relative ion leakage was checked following the method of Clarke et al. (2004). For the above assays, each data point is the average of three replicates. At least three experiments were performed, and the results are consistent. The result from one set of experiments is presented here.

### Oxidative Enzyme Activity Measurements

The leaves of 4-week-old rice seedlings were dehydrated for 2 h, and then homogenized in a solution of 50 mM sodium phosphate buffer (pH 7.8) containing 1% polyvinylpyrrolidone and 10 mM β-mercaptoethanol in an ice-cold mortar. After centrifugation (13,000 × *g*, 15 min) at 4°C, the supernatant was used to identify SOD, POD and CAT activity levels. U min^-1^ mg^-1^ protein was represented the enzyme activity of SOD, POD, and CAT.

The ability to inhibit the photochemical reduction of NBT chloride was used for the determination of the total SOD activity as described by Beauchamp and Fridovich (1971). The reduction of NBT by 50% of the quantity of enzyme required was defined as one unit of SOD activity.

The activity of POD was determined as described by Maehly and Chance (1954). Three milliliter of reaction mixture contained 30 μl enzyme extract, 5.4 mM guaiacol, 50 mM sodium acetate buffer (pH 5.6), and 15 mM H_2_O_2_. The oxidation of guaiacol to tetraguaiacol was contributed to the increase in absorbance monitored at 470 nm. A 0.01 absorbance increase per min at 470 nm was defined as one unit of POD activity.

The activity of CAT was measured following the method of Cakmak and Marschner (1992) by determining the rate of H_2_O_2_ disappearance at 240 nm. Three milliliter of reaction mixture contained 30 μl enzyme extract, 10 mM H_2_O_2_ and 50 mM phosphate buffer (pH 7.0). A 0.01 absorbance decrease per min at 240 nm was defined as one unit of CAT activity.

For each enzyme’s activity, the data points are the average of three replicates. Three experiments were performed, and the results are consistent. The result from one set of experiments is presented here.

### Salt and Osmotic Tolerance Assays

For salt tolerance assays, 2-week-old seedlings grown on half-strength MS agar medium were transferred into half-strength MS liquid medium for 2 weeks growth and then transferred into half-strength MS liquid medium supplemented with 175 mM NaCl and incubated at 22°C under long-day conditions for 2 days. After 2 days of NaCl treatment, the seedlings were transferred into half-strength MS liquid medium for 7 days recovery.

For PEG tolerance assays, 2-week-old seedlings grown on half-strength MS agar medium were transferred into half-strength MS liquid medium for 2 weeks of growth and then transferred into half-strength MS liquid medium supplemented with 25% PEG6000 (m/v) and incubated at 22°C under long-day conditions for 4 days. After 4 days of PEG treatment, the seedlings were transferred into half-strength MS liquid medium for 7 days recovery.

The transgenic rice and control seedlings were transplanted in the same pot for NaCl and PEG treatments. Three independent pots repeated at the same time and a representative result displayed in the manuscript. Three independent experimental replications were conducted.

### Real-Time RT-PCR Analysis

For the real-time RT-PCR analysis, the same method was used as described in our previous studies (Jiang et al., 2012, 2014). We conducted three independent experiments (three biological replications and three technological replications in every independent experiment) and one representative result was displayed. All of the primer sequences used in real-time RT-PCR analysis were listed in Supplementary Table 1.

### Statistical Analysis

Statistically significant differences (^∗^*P* < 0.05) based on the Student’s test computed by the SigmaPlot10.0. Data are the means ± SE of three independent experiments (3 biological replications and three technological replications in every independent experiment).

## Results

### Constitutive Expression of *AtWRKY57* in Transgenic Rice Lines

**FIGURE 1:**
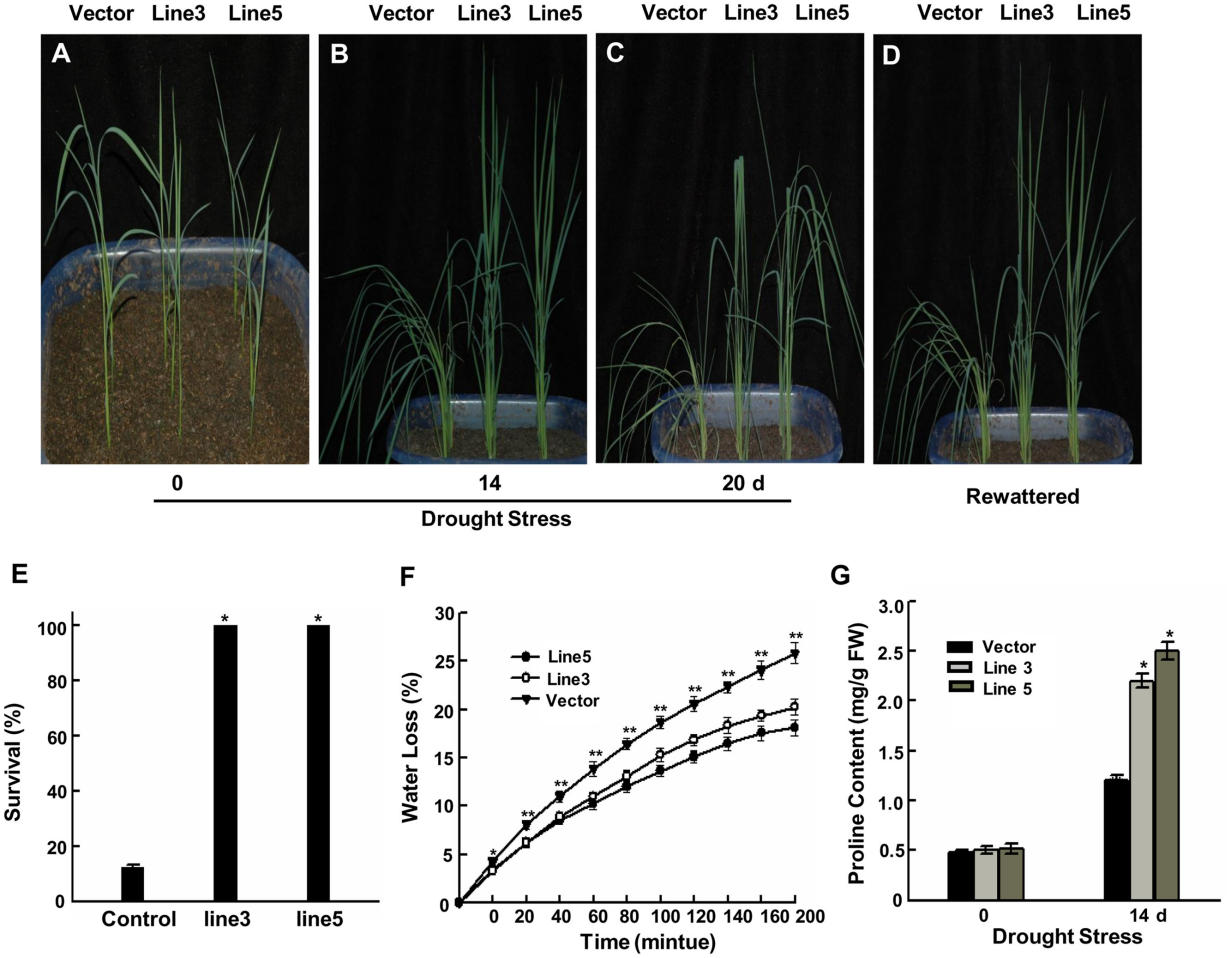
**Improved drought tolerance in *AtWRKY57* transgenic rice. (A)** Before drought treatment. **(B)** Drought for 14 days. **(C)** Drought for 20 days. **(D)** Recovery for 7 days after 20 days drought treatment. Drought stress was imposed on 4-week-old T3 transgenic seedlings in greenhouse. Drought experiments were repeated three times and at least 40 plants for each individual lines were used in each repeated experiment and one representative picture was shown. **(E)** Survival rate after 20 days drought stress. Values are mean ± SE (*n* = 40 plants, ^∗^*P* < 0.05). **(F)** Rate of water loss by detached leaves from control and transgenic plants. Values are the mean ± SE (*n* = 6 plants, ^∗∗^*P* < 0.01). **(G)** Proline content in the leaves of 4-week-old transgenic and control plants with or without drought treatments. Values are the mean ± SE of three independent experiments (^∗^*P* < 0.05). FW, Fresh weight.

In our previous study, we confirmed that overexpression of *AtWRKY57* significantly enhanced drought tolerance in *Arabidopsis* (Jiang et al., 2012). To explore whether *AtWRKY57* plays an important role in improving the agronomic traits through gene manipulation approaches, we introduced this gene to rice. More than 20 transgenic lines were generated and five lines were randomly selected to check *AtWRKY57* expression by northern blotting (Supplementary Figure 1). And then two lines, Line 3 and Line 5, were chosen for further analysis (Supplementary Figure 1). There were no significant differences in morphology between the control and transgenic plants (**Figure 1A**).

### Improved Drought Tolerance in *AtWRKY57* Transgenic Plants

The transgenic lines and control seeds were germinated simultaneously on half-strength MS agar medium containing 2% sucrose with or without hygromycin at 100 μg/ml and then planted in soil after 1 week. Four-week-old plants were treated with natural drought stress (not supplied with water). The control plants showed wilting symptoms 6 days before the transgenic lines. After 14 days treatment, the transgenic plants did not display any drought-stress symptoms, while the wild-type plants exhibited severe drought symptoms (**Figure 1B**). Up to 20 days of treatment, the control showed obvious drought-stress symptoms (**Figure 1C**). When plants were re-watered, only 12.3% of control plants were survived and most of them never recovered; however, all of the transgenic rice plants survived (**Figures 1D,E**). These results suggested that these transgenic rice plants acquired significantly improved drought tolerance. Soil moisture contents and their dynamics showed in Supplementary Figure 2.

Transpiration water loss is an important factor related to drought tolerance. Flag leaves were detached and the changes of fresh weight were determined over a 200-min period to assess the water loss rate of transgenic and control plants. A slower water loss rate was displayed in the transgenic lines’ leaves than the control’ (**Figure 1F**). The reduced water loss rate is favorable for an increased drought tolerance in the transgenic lines. In response to drought stress, stomata often close to limit water loss by transpiration. Given that water loss rate were lower in two transgenic lines than in control plants, we further investigated whether stomata density and/or stomata aperture affects this progress. White nail polish blotting was used to count the stomata density and measure stomata aperture. The ratio of stomatal width to length indicated the degree of stomatal closure. The results showed that the stomata density didn’t displayed significant difference between control and two transgenic plants leaf adaxial surface (Supplementary Figures 3A,B). However, two transgenic lines’ stomata showed more quick closure than control’ under dehydration treatments (Supplementary Figure 3C). These results suggest that more quick closure of stomata in two transgenic lines result in the lower water loss rate, which may be critical for transgenic plants to adapt to drought stress.

The accumulation of proline in plant is associated with adaptation to environmental stress through metabolic adjustments (Ábrahám et al., 2003). We also checked the proline contents of transgenic and control plants under normal growth and drought-stress conditions to characterize the physiological basis for the improved stress tolerance. No differences in the proline contents were observed in the leaves of transgenic and control plants under normal conditions (**Figure 1G**). However, under drought conditions, transgenic plants began to accumulate proline after 14 days and further accumulated an up to fourfold higher proline content compared with the levels prior to drought stress, whereas control plants showed a low increase in proline. This result demonstrates that the proline accumulation corresponded to the increased drought tolerance of transgenic plants.

### Decreasing the ROS Damage in *AtWRKY57* Transgenic Plants

**FIGURE 2:**
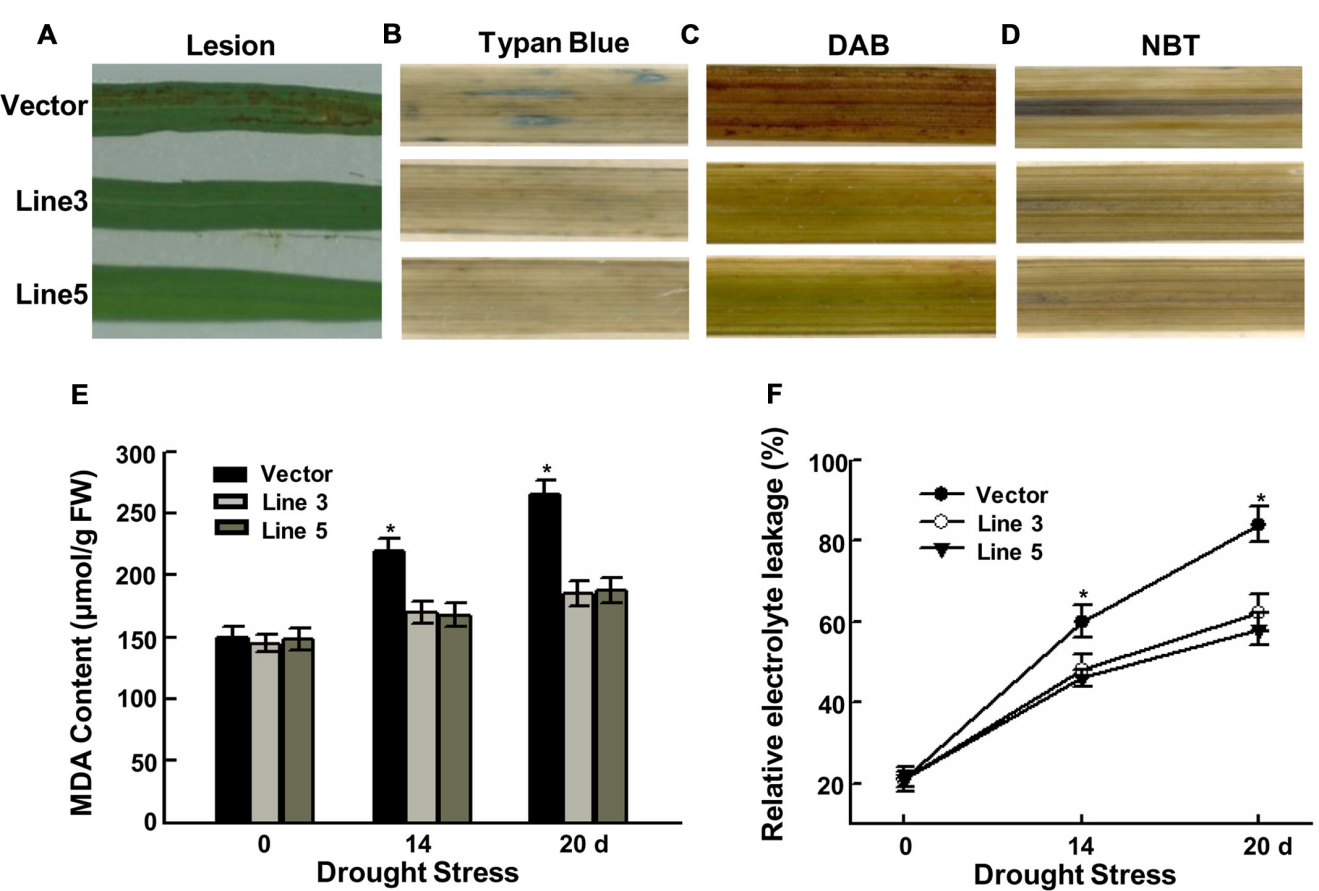
**Decreased the ROS damage in transgenic rice.** Phenotype of brownish lesions **(A)**, Typan blue staining **(B)**, DAB staining **(C)** and NBT staining **(D)** after 14 days drought stress. **(E)** MDA content in the leaves of control and transgenic plants after 0, 14, and 20 days drought stress. Values are the mean ± SE of three independent experiments (^∗^*P* < 0.05). FW, Fresh weight. **(F)** Relative electrolyte leakage in the leaves of control and transgenic plants after 0, 14, and 20 days drought stress. Values are the mean ± SE of three independent experiments (^∗^*P* < 0.05).

Leaves of control plants began to produce brownish lesions after 14 days of drought stress (**Figure 2A**). In contrast, none of the transgenic plants exhibited lesion formation grown under the same conditions. Lesion formation was accompanied by significant trypan blue staining that indicates cell death in the control leaves (**Figure 2B**).

Stress usually causes damage via oxidative damage in plants including the generation of ROS, represented as H_2_O_2_ and superoxide (Zhu, 2001; Mittler, 2002; Xiong and Zhu, 2002). As activation of *AtWRKY57* enhanced the drought tolerance of transgenic rice plants, we further determined whether *AtWRKY57* is involved in drought tolerance via ROS detoxification. Transgenic rice and control seedlings were subjected to DAB staining and NBT staining to detect H_2_O_2_ and superoxides in their leaves. After 14 days of drought stress, the transgenic plants had very few brown H_2_O_2_ and superoxide spots within the leaf segments, whereas more than half of the leaf area of the control plants became brown (**Figures 2C,D**). The leave segments of control plants displayed more brown areas than compared transgenic plants. These results confirmed that over-expressing *AtWRKY57* in rice could efficiently remove the H_2_O_2_ and superoxide produced during drought stress.

Malondialdehyde, acting as a biomarker for lipid peroxidation, is an effect of oxidative damage deriving from decomposition product of polyunsaturated fatty acid hydroperoxides. The MDA contents in transgenic lines and controls were similar under normal growth conditions, but there was a significant difference after drought stress. Then, the MDA contents of two *AtWRKY57* over-expressing lines were significantly lower than those of control plants (**Figure 2E**).

Electrolyte leakage, an indicator of membrane damage, was also measured following drought stress. The results showed that the leaves of two *AtWRKY57* over-expressing lines exhibited significantly lower electrolyte leakage levels, compared to those of control leaves (**Figure 2F**). After 14 days of drought stress, more than 60% of the ions leaked from cells in control plants, whereas the ion leakage of *AtWRKY57* over-expressing lines was less than 50%.

Overall, these results indicated that the over-expressing *AtWRKY57* gene in rice increased the tolerance to drought stress by decreasing ROS damage.

### The Enhanced ROS-Scavenging Ability and High-Level Expression of Oxidative Enzyme Genes in *AtWRKY57* Transgenic Plants

**FIGURE 3:**
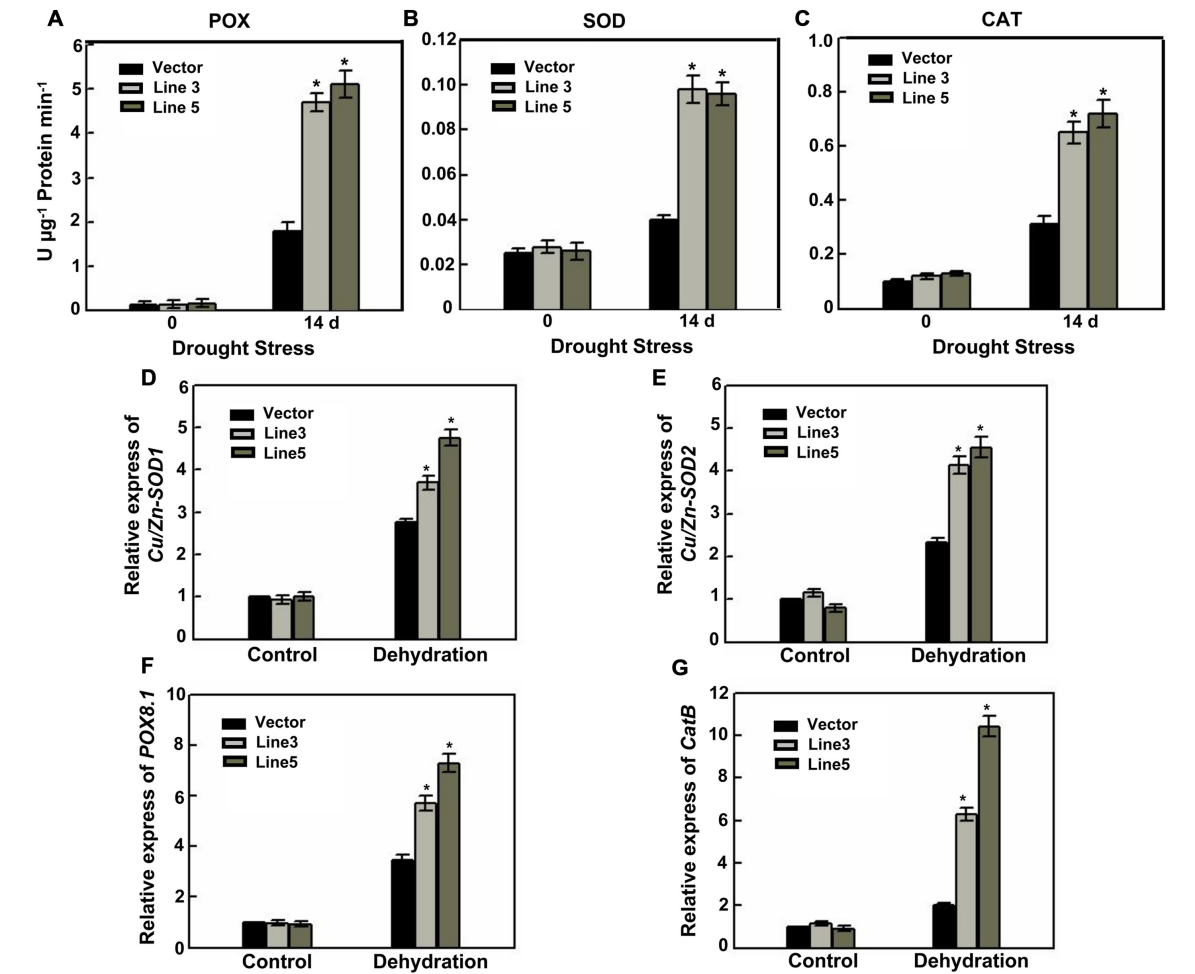
**Enhanced the ROS-scavenging ability and the expression of oxidative enzymes genes in transgenic rice. (A–C)** POX, SOD, and CAT activities in the leaves of 4-week-old transgenic and control plants before and after drought stress. Values are the mean ± SE of three independent experiments (^∗^*P* < 0.05). FW, Fresh weight. Relative expression of oxidative enzymes genes *Cu/Zn-SOD1* **(D)**, *Cu/Zn-SOD2* **(E)**, *POX8.1* **(F)**, and *CatB* **(G)** in the leaves of 4-week-old transgenic and control plants before and after drought stress. Values are the mean ± SE of three independent experiments (^∗^*P* < 0.05).

A decreased cell viability and even cell death was resulted from the over-accumulation of ROS; therefore, scavenging ROS avoids or alleviates the harmful effects on plant under stress conditions. In the ROS-scavenging mechanisms of plants, POD, SOD, and CAT are key enzymes (Mittler, 2002; Xiong and Zhu, 2002; Apel and Hirt, 2004), and are involved in the H_2_O_2_ elimination. Following drought stress, the enzyme activities of seedlings were subjected to measurement. Under normal growth conditions, POD, SOD, and CAT activity levels were not different; however, after 14 days of drought stress, the activities of the antioxidative enzymes were all significantly enhanced in the *AtWRKY57*-overexpressing plants compared with those in the control plants (**Figures 3A–C**). These results suggested that over-expression of *AtWRKY57* gene may enhance the ROS-scavenging ability, which decreases ROS damage.

To test whether drought stress modifies transcript levels, the expression levels of several antioxidant genes were measured. Consistent with the increase of antioxidative enzymes activities, control and two transgenic plants up-regulated the transcript levels of *OsCAT B, OsCu/Zn-SOD1, OsCu/Zn-SOD2* and *OsPOD* in response to drought stress, with a greater increase in the transgenic plants (**Figures 3D–G**). This was enhancing the capacity to decompose H_2_O_2_ and superoxide in the leaves.

### High-Level Expression of Stress-Response Genes in *AtWRKY57* Transgenic Plants

**FIGURE 4:**
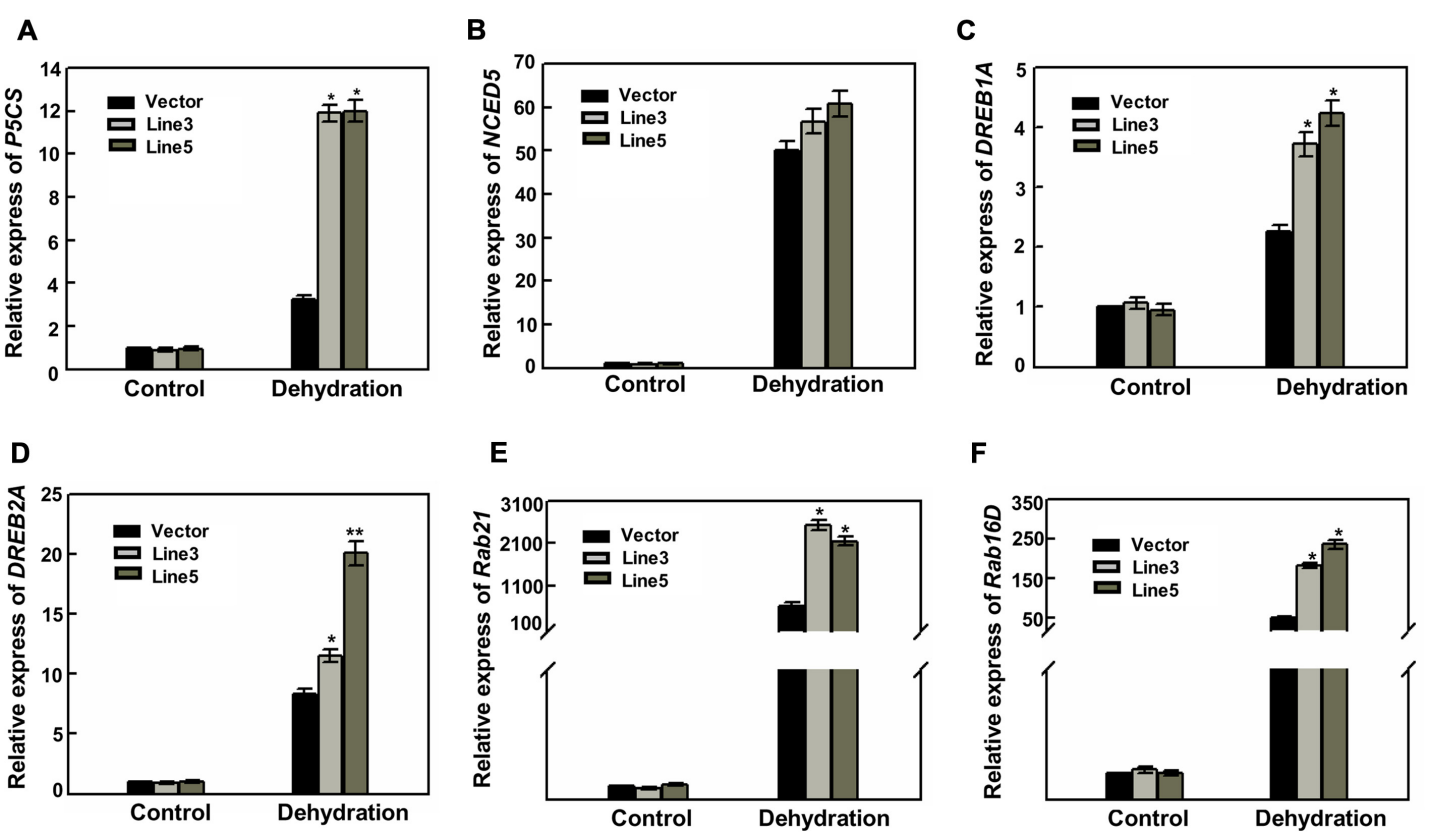
**High level expression of stress-responsive genes in transgenic rice. (A–F)** Relative expression levels of *P5CS, NCED5, DREB1A, DREB2A, Rab21* and *Rab16D* in the leaves of 4-week-old transgenic and control plants before and after drought stress. Values are the mean ± SE (^∗^*P* < 0.05) of three independent experiments.

To better understand the mechanisms of drought tolerance conferred by over-expressing *AtWRKY57*, the expressions of several stress-related genes were investigated. As shown in **Figure 4A**, the expression levels of a pyrroline-5-carboxylate synthesis gene (*OsP5CS*; Igarashi et al., 1997) were strongly induced in transgenic lines under drought stress compared with those in control plants. This higher expression level of *OsP5CS* was consistent with the higher proline content in two *AtWRKY57* overexpressing lines (**Figure 1G**). However, the expression level of an ABA synthesis gene (*OsNCED5*) was not significantly different in transgenic lines and control plants after drought stress (**Figure 4B**). Dehydration-responsive element-binding (DREB) transcription factors play key important roles in plant-stress responses. DREB proteins encoding by *OsDREB1A* and *OsDREB2A* were strongly up-regulated in drought stress, with a greater increase in the transgenic plants compared to the control plants (**Figures 4C,D**). We also checked another two well-characterized drought resistance-related genes (*OsRab21* and *OsRab16D*), and found that they were significantly affected by water stress. Their expression levels were obviously higher in transgenic plants than in control after drought treatment (**Figures 4E,F**).

These results indicated that over-expressing *AtWRKY57* gene in rice may enhance the expression of some stress-response genes and finally increase the tolerance to drought stress.

### Improved NaCl and PEG Tolerance in *AtWRKY57* Transgenic Plants

**FIGURE 5:**
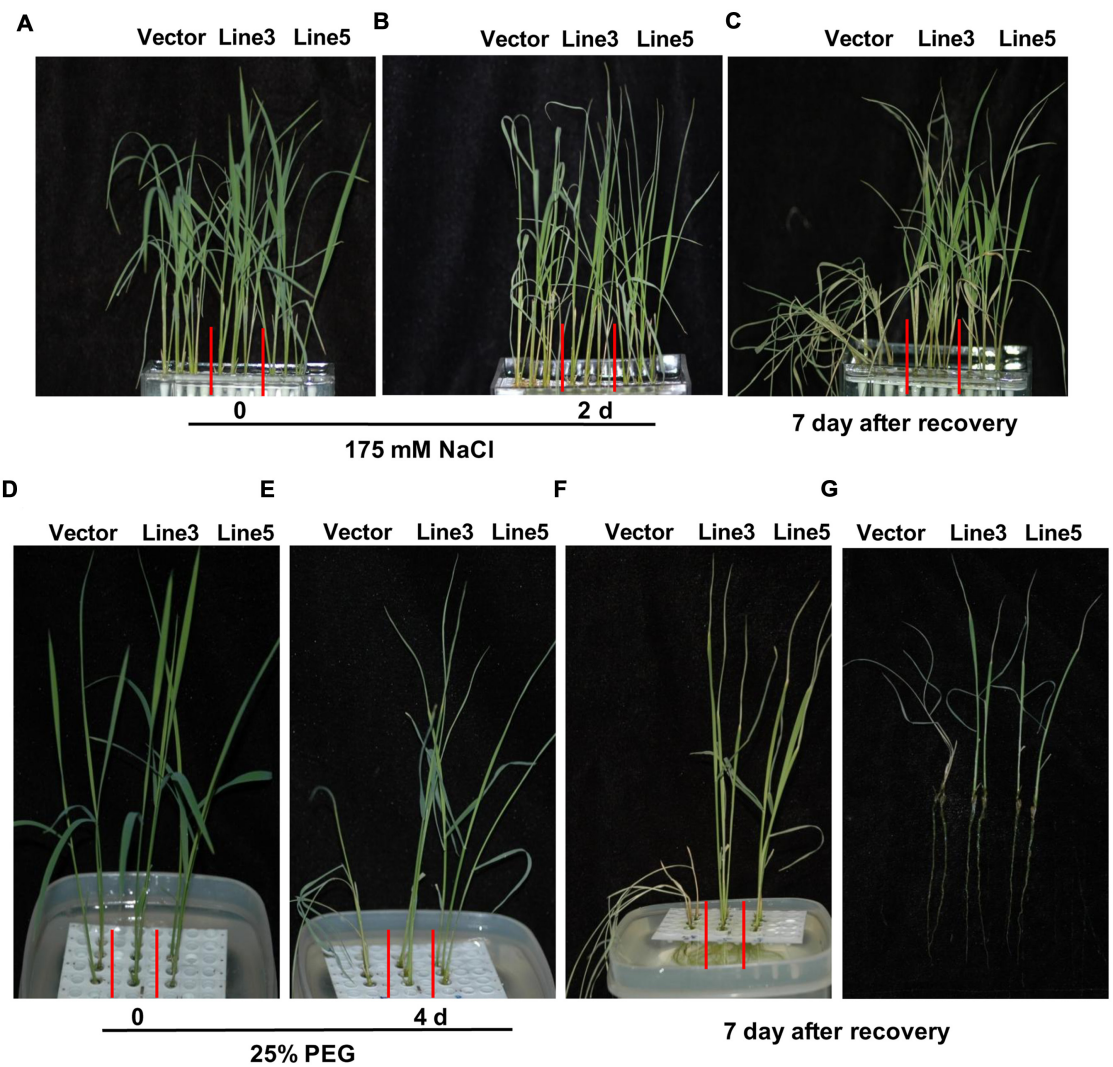
**Improved NaCl and PEG tolerance in transgenic rice. (A)** Before NaCl treatment. **(B)** NaCl treatment for 2 days. **(C)** Recovery for 7 days after 2 days NaCl treatment. **(D)** Before PEG treatment. **(E)** PEG treatment for 4 days. **(F,G)** Recovery for 7 days after 4 days PEG treatment. NaCl and PEG stress was imposed on 4-week-old T3 transgenic seedlings under water culture conditions in greenhouse. Experiments were repeated three times and at least 30 plants for each individual lines were used in each repeated experiment and one representative picture was shown.

Our results revealed that the constitutive expression of *AtWRKY57* enhanced the drought tolerance in rice (**Figure 1**). Given the function of WRKY-type regulators in abiotic stress, we further explored the functions of *AtWRKY57* in NaCl and PEG stress conditions. We tested the survival rates of transgenic and control plants on MS medium additionally added with 175 mM NaCl. The control plants displayed more severe phenotype, including leaf curves and dehydration, than the transgenic lines after 2 days of NaCl treatment (**Figures 5A,B**). When plants were recovered in fresh MS medium, none of the control plants survived but most of the transgenic lines reversed (**Figure 5C**; Supplementary Figure 4). We also tested the survival rate of transgenic lines and control plants on MS medium supplemented with 25% PEG6000. The control plants showed more severe phenotype than transgenic lines after 4 days PEG treatments (**Figures 5D,E**). When plants were recovered in fresh MS medium, none of the control plants survived, but all of the transgenic lines lived (**Figures 5F,G**; Supplementary Figure 5).

These results showed that consecutively expressing *AtWRKY57* enhanced not only drought tolerance but also the NaCl and PEG stress tolerance in rice.

## Discussion

Combination of abiotic and biotic stresses used to limit the production of crop. Drought severely restricts crop production as the most important abiotic stress (Boyer, 1982; Rockstrom and Falkenmark, 2000). In a previous study, we confirmed that over-expressing *AtWRKY57* significantly conferred drought tolerance in *Arabidopsis* (Jiang et al., 2012). These results suggested that *AtWRKY57* may improve crops’ drought adaptability using gene manipulation. In this study, we evaluated the role of *AtWRKY57* in transgenic rice after drought stress.

The drought-tolerance phenotype of *AtWRKY57* transgenic rice plants were the result of a collection of physiological indexes observed in the over-expressing plants. *AtWRKY57* overexpressing plants displayed higher survival rates most likely because the water loss was reduced in these plants compared to control plants under drought conditions (**Figure 1F**). *P5CS*, catalyzing proline biosynthesis, is critical for the increasing of osmotolerance. Drought, salt, and abscisic acid induce the expression of *OsP5CS* and the conferred osmotolerance is reuslting from an up-regulated expression of *OsP5CS* which increases proline content in transgenic plants (Xiang et al., 2007). The significantly higher transcript levels of *OsP5CS* were consistent with the high proline content in transgenic plants after drought stress (**Figures 1G** and **4A**). These results confirmed that the transgenic plants’ adaptation to drought stress was associated with mechanisms of dehydration avoidance through proline metabolic adjustments. Programmed cell death (PCD) and lesion formation in some lesion mimic mutants, such as *lsd1*, was mainly caused from the elevated levels of extracellular superoxide (Jabs et al., 1996). We ovserved high levels of superoxide, H_2_O_2_ and cell death in control plants than in transgenic lines. We believe that the lesion formation in control plants results from their reduced capability to detoxify ROS compared with transgenic plants (**Figure 2A**). These results are similar to those of Yang et al. (2004), who reported that salicylic acid-deficient transgenic rice contains elevated levels of superoxide and H_2_O_2_ and exhibits spontaneous lesion formation in an age- and light-dependent manner.

Drought or salt-stress conditions promoted the accumulation of ROS in plants. MDA is often considered as a reflection of cellular membrane degradation or dysfunction and is also an important intermediary agent in ROS scavenging. Thus, high level of MDA causes PCD and induces toxicity to plant cells (Apel and Hirt, 2004; Hou et al., 2009). High ability of ROS-scavenging enzymes decreased over-accumulated ROS levels which induces PCD in plants (Mittler, 2002; Apel and Hirt, 2004; Chaves and Oliveira, 2004; Farooq et al., 2009; Hou et al., 2009). In *AtWRKY57* transgenic plants, lower levels of PCD, DAB and NBT staining, MDA content and relative electrolyte leakage were detected (**Figures 2B–F**), but increased SOD, POD, and CAT activity levels (**Figures 3A–C**) and elevated oxidative enzyme genes’ transcript levels (**Figures 3D–G**) were detected after drought stress, demonstrating that they were better protected from oxidative damages through the enhanced capability to scavenge ROS.

The transcript levels for several stress-tolerant genes were more elevated in *AtWRKY57* transgenic rice than in control plants under drought-stress conditions (**Figure 4**). It is interesting that the relative transcript levels of *OsNCED5* and the ABA content were not significantly changed (**Figure 4B**; Supplementary Figure 6) in *AtWRKY57* transgenic plants under drought-stress conditions, which may demonstrate that there were different regulatory mechanisms in transgenic *Arabidopsis* and transgenic rice. Our previous study revealed that the activated expression of *AtWRKY57* conferred *Arabidopsis* transgenic plants drought tolerant by elevating the ABA contents through directly binding the promoter sequence of *AtNCED3* (Jiang et al., 2012). In this study, we found that the enhanced capability to scavenge ROS was important for *AtWRKY57* overexpressing transgenic rice plants to tolerate drought stress (**Figures 2** and **3**). Interestingly, there are increasing studies demonstrated that the same gene may have different regulatory functions and/or mechanisms when overexpressed in different plants species, such as in rice, cotton and *Arabidopsis*. For example, *OsWRKY45* overexpressing transgenic rice showed sensitivity to drought stress (Tao et al., 2011); however, heterologous overexpression of *OsWRKY45* in *Arabidopsis* conferred plants drought tolerant mainly resulting from the reduction of transpiration rate (Qiu and Yu, 2008). Overexpression of *OsSNAC1* enhanced drought tolerance of transgenic rice plants by targeting genes that control ROS homeostasis and stomatal closure (Yu et al., 2013), whereas overexpressing *OsSNAC1* rendered transgenic cotton plants more drought tolerance by reducing transpiration rate and enhancing root development (Liu et al., 2014). Heterologous expression of the *AtDREB1A* gene in peanut conferred transgenic plants drought and NaCl tolerance by upregulating proline synthesis to better osmotic adjustments (Sarkar et al., 2014), while the *AtDREB1A* transgenic *Arabidopsis* enhanced drought by activating some stress-related genes expression (Liu et al., 1998). Thus, it’s possible that there may be diversified regulatory functions and/or mechanisms for one protein to regulate different physiological processes in different species under stress conditions.

WRKY transcription factors belong to a large family that functions under a variety of abiotic stresses. Our results provided evidences that overexpressing *AtWRKY57* also increased the tolerance to salt and PEG stresses (**Figures 1** and **5**), demonstrating that this is a potential candidate gene for crop improvement. Recently, several studies confirmed that overexpression of some stress-related genes may enhance drought tolerance in rice (Dubouzet et al., 2003; Park et al., 2005; Chen et al., 2008; Hou et al., 2009; Huang et al., 2009; Cui et al., 2011; Gao et al., 2011; Yang et al., 2012; Zou et al., 2012). However, a persistent problem is that the constitutive over-expression of stress-related genes often result in abnormal development and thus reduces crop productivity (Kasuga et al., 1999; Hsieh et al., 2002; Dubouzet et al., 2003; Nakashima et al., 2007; Priyanka et al., 2010). The improvement in drought tolerance should be perfectible without limitation in plant growth and production (Cattivelli et al., 2008). Yu’s study confirmed that the heterologous expression of *AtEDT1/HDG11* in rice significantly improved its drought tolerance and also simultaneously increased the grain yield under both normal and drought-stress conditions (Yu et al., 2013). In our study, *AtWRKY57* transgenic plants underwent normal development compared with controls (**Figure 1A**), but we have not statistically analyzed the effects on productivity. Further studies should focus on the grain yield of transgenic plants under drought-stress conditions.

## Author Contributions

YJ designed and performed experiments, interpreted data, and wrote the article. DY designed experiments, and edited the article. YQ and YH interpreted data and edited the article. Both authors read and approved the final article.

## Conflict of Interest Statement

The authors declare that the research was conducted in the absence of any commercial or financial relationships that could be construed as a potential conflict of interest.
