# Supplementary material for: Heterologous Expression of AtWRKY57 Confers Drought Tolerance in Oryza sativa
Source: Front Plant Sci. 2016 Feb 11;7:145. doi: 10.3389/fpls.2016.00145 (PMC4749717; doi:10.3389/fpls.2016.00145)

**Supplementary Material**

**Heterologous expression of *AtWRKY57* confers drought tolerance in *Oryza sativa***

**Yanjuan Jiang^1^, Yuping Qiu^2^, Yanru Hu^1^, and Diqiu Yu^1^***

***Correspondence：**Corresponding Author: Diqiu Yu e-mail:[ydq@xtbg.ac.cn](mailto:ydq@xtbg.ac.cn)

**Supplementary Fig. 1 Northern blot analysis of transgenic lines.** The experiment was repeated three times, and a typical RNA gel blot is presented.


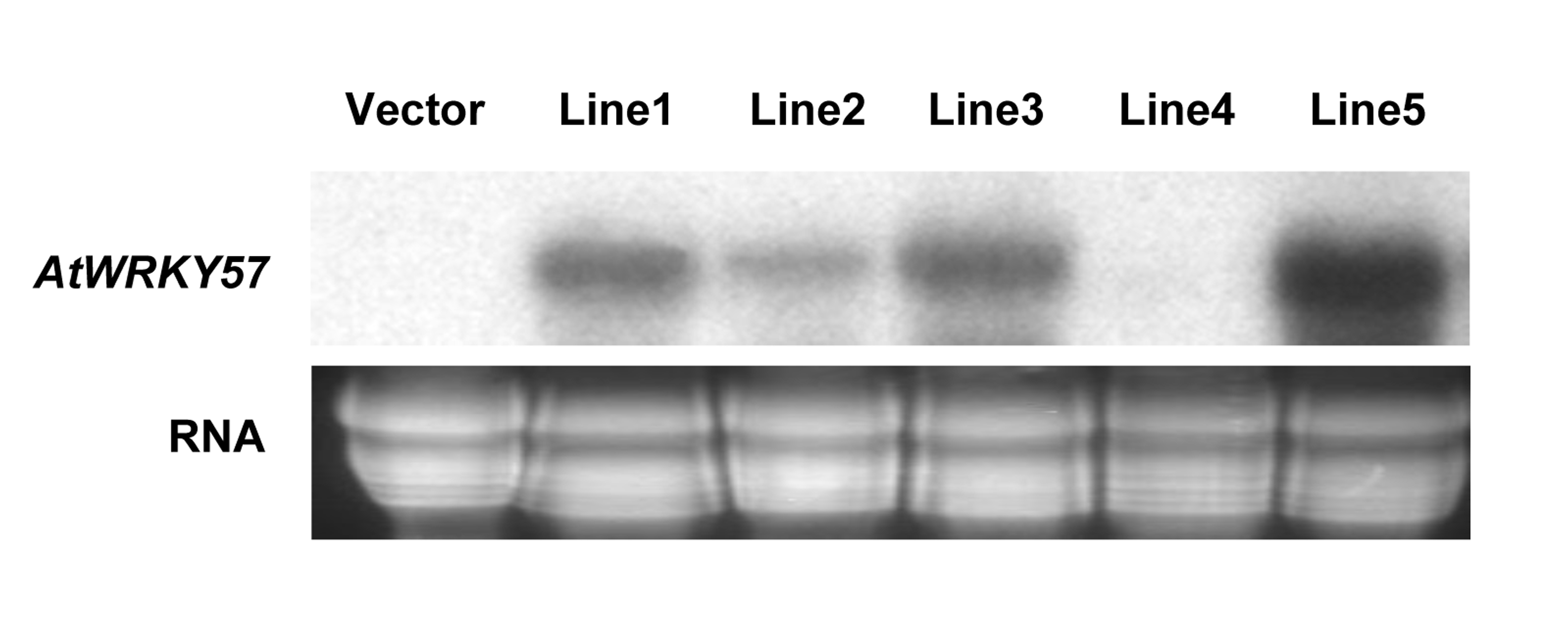


**Supplementary Fig. 2 Soil moisture content before and after drought stress**


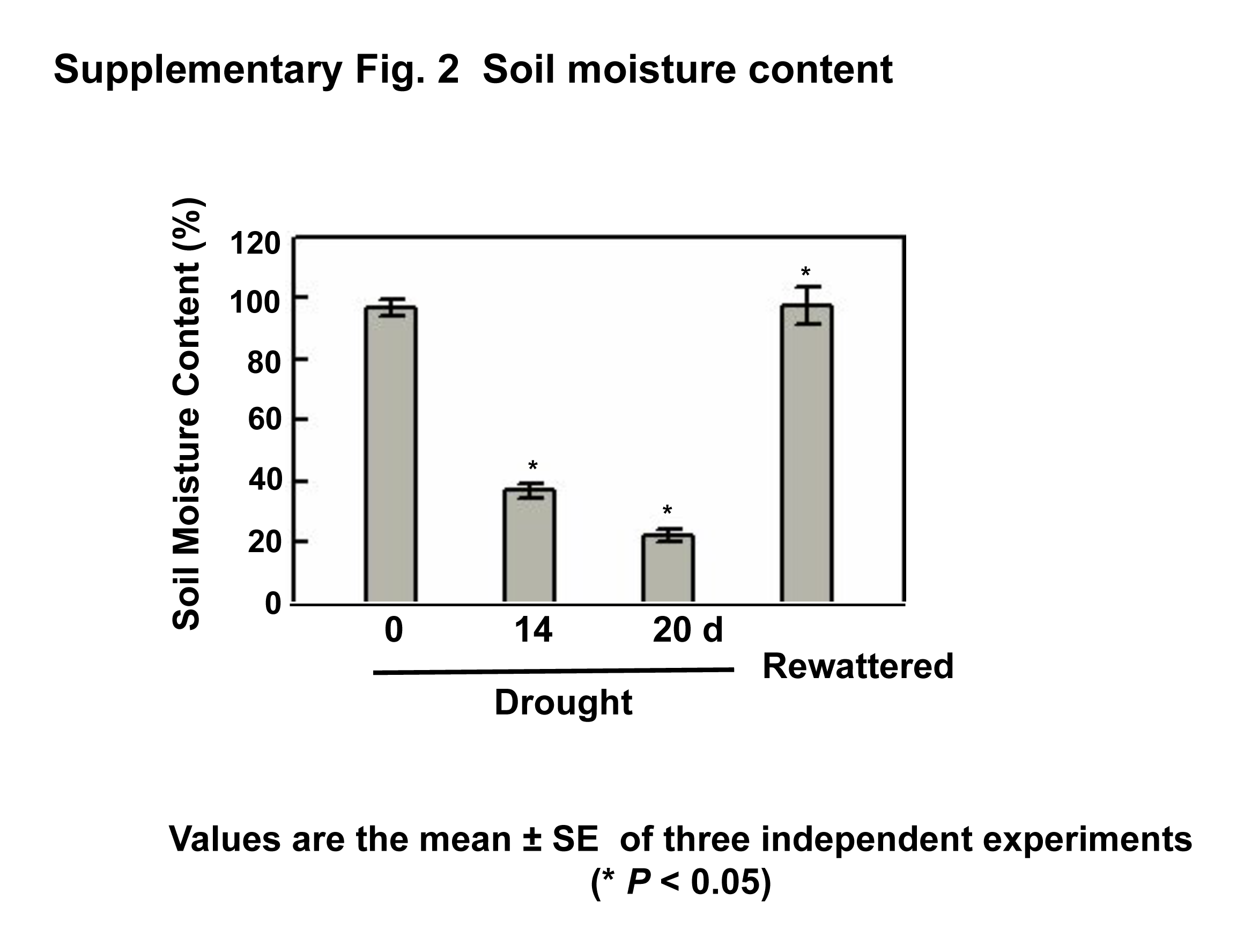


**Supplementary Fig. 3 Stomata density and aperture**

**
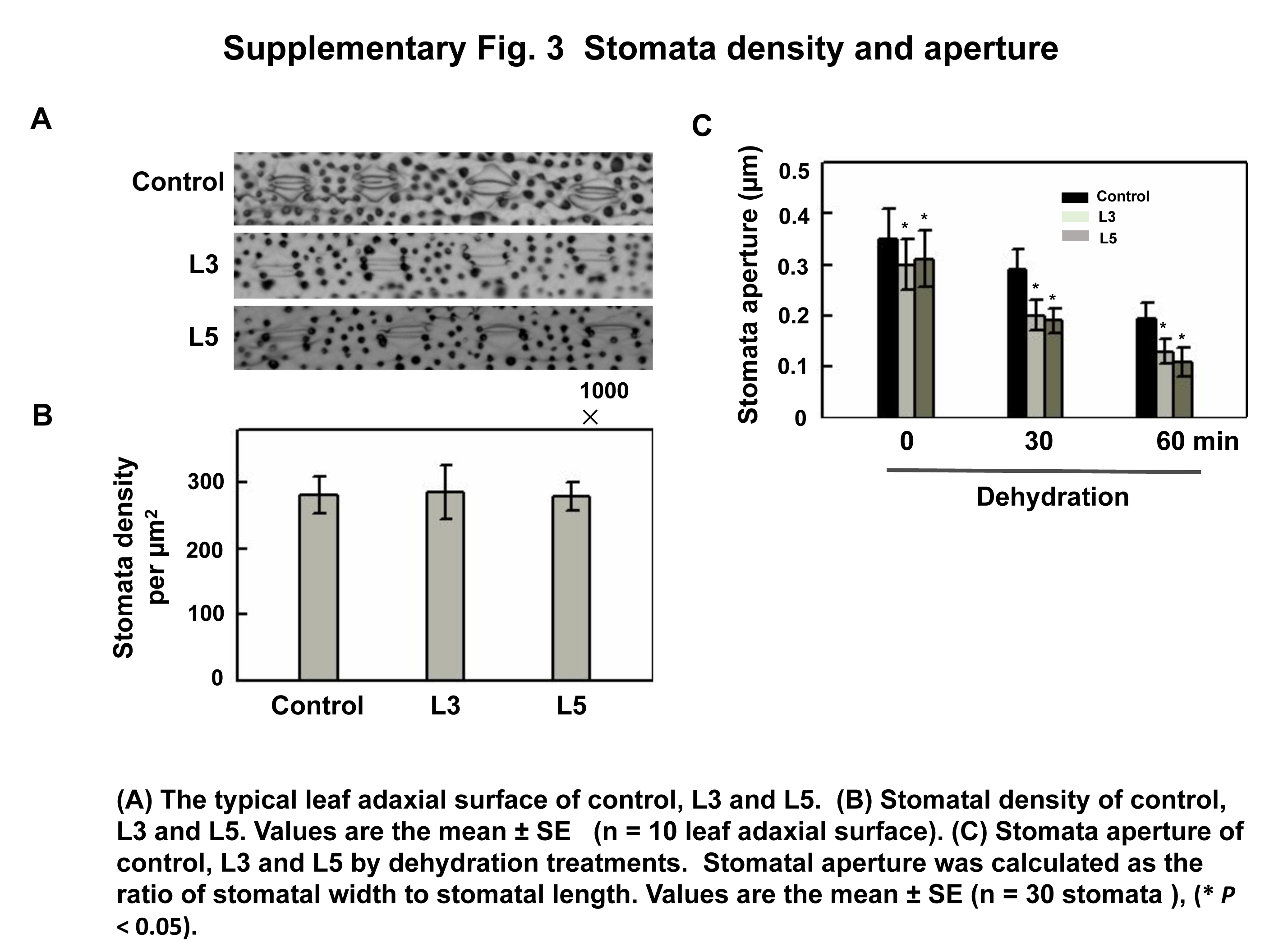
**

**Supplementary Fig. 4 Survival rate after NaCl treatment.** Values are the mean (n = 30 plants).


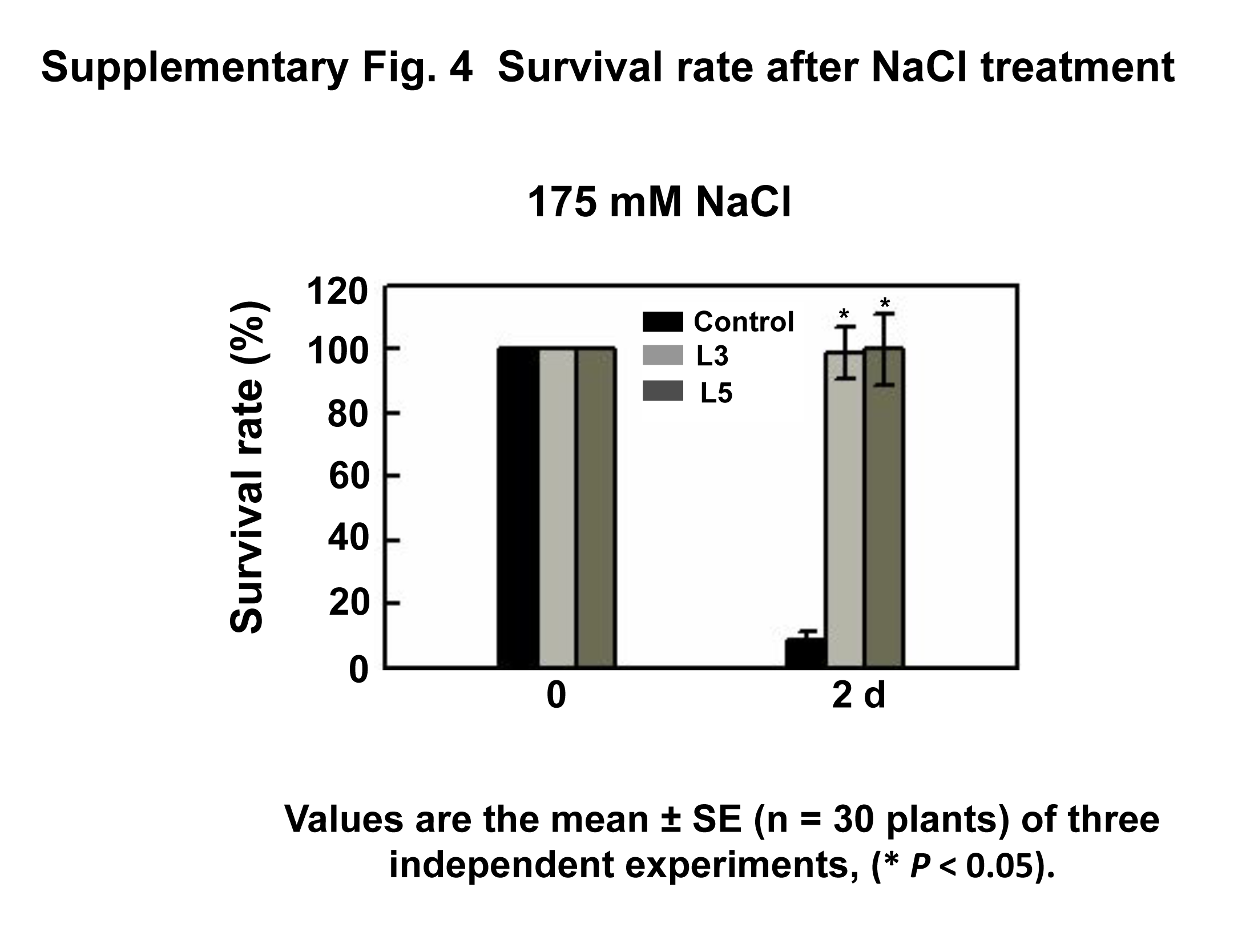


**Supplementary Fig. 5 Survival rate after PEG treatment.** Values are the mean (n = 30 plants).


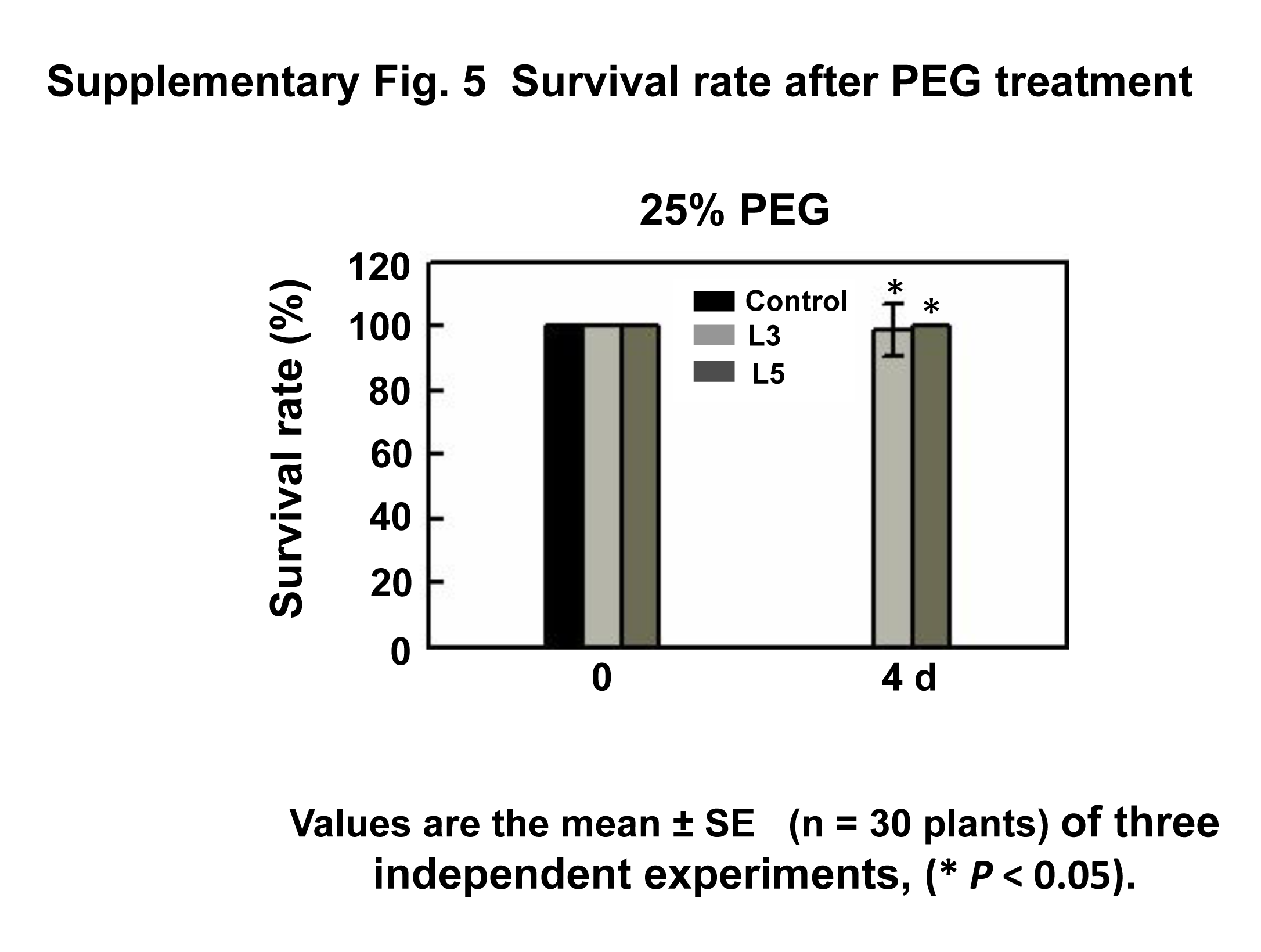


**Supplementary Fig. 6 ABA content before and after drought stress**


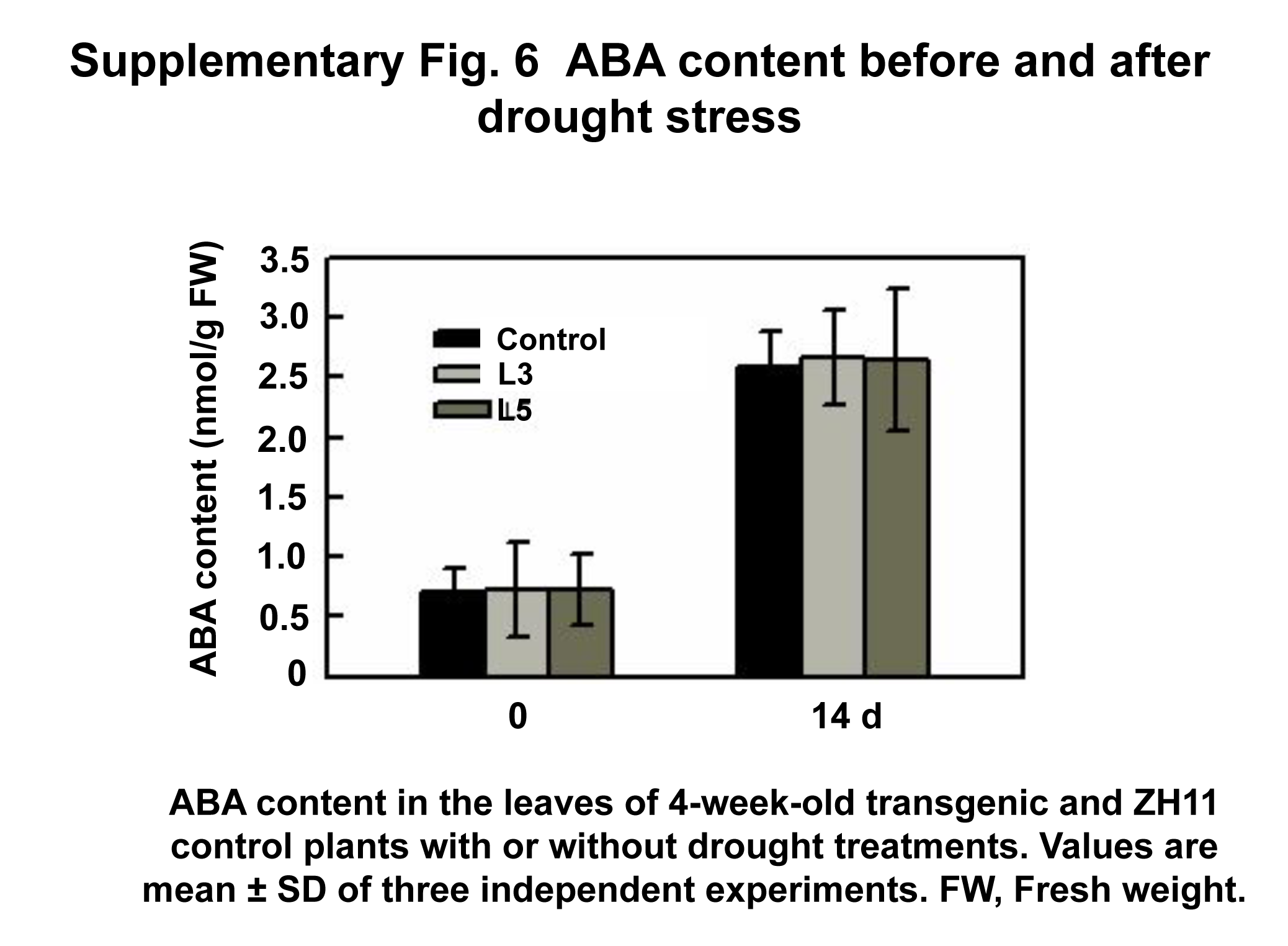


**Supplementary Table 1. Primer sequences used in real-time RT-PCR**


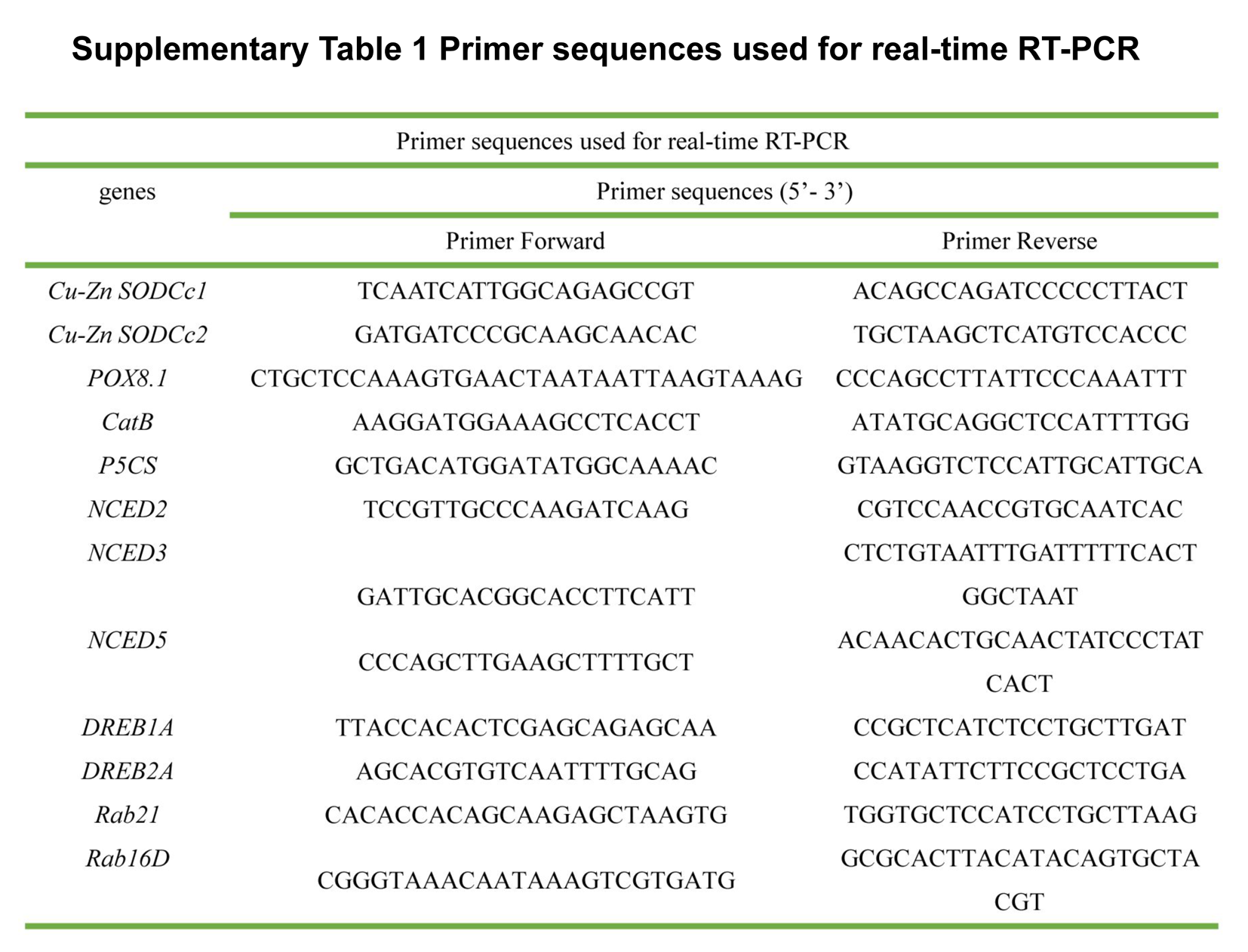

Supplement: Supplementary file 1 [file Data_Sheet_1.DOC]
